# Supplementary material for: SsNEP2 Contributes to the Virulence of Sclerotinia sclerotiorum
Source: Pathogens. 2022 Apr 7;11(4):446. doi: 10.3390/pathogens11040446 (PMC9026538; doi:10.3390/pathogens11040446)
Supplement: Supplementary file 1 [file pathogens-11-00446-s001.zip › pathogens-1636551-supplementary.pdf]

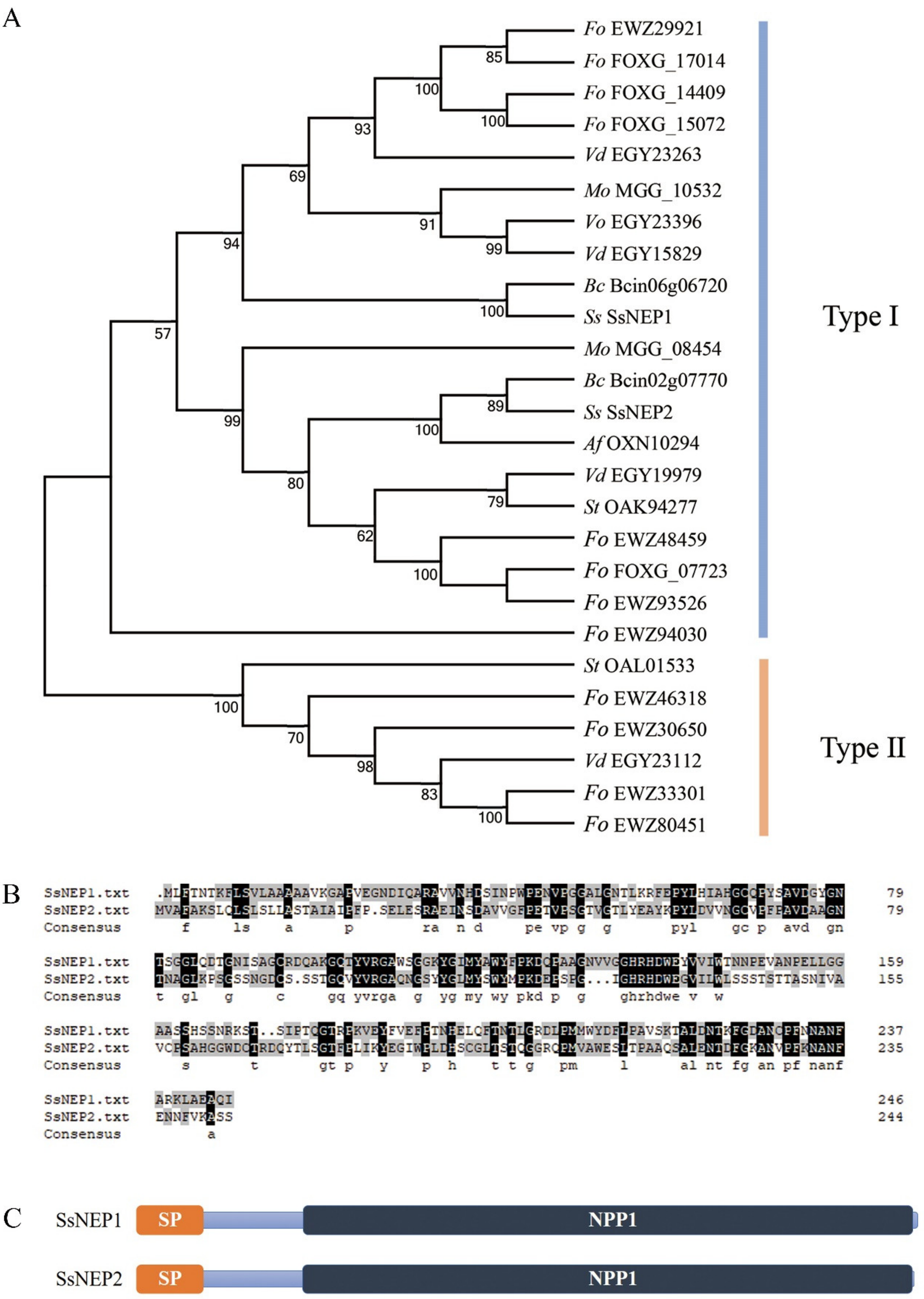

Supplementary Figure S1. Bioinformatics analysis of *Ss*NEP1 and *Ss*NEP2 protein. (A) Phylogenetic tree of *Ss*NEP1 and *Ss*NEP2 was constructed based on amino acid sequences from different fungi. Data downloaded from EnsemblFungi (http://fungi.ensembl.org/index.html). (B) Alignment of amino acid sequences of *Ss*NEP1 and *Ss*NEP2. (C) Protein domain structure analysis. Domain architectures were identified using Pfam database (https://pfam.xfam.org/). SP, signal peptide.

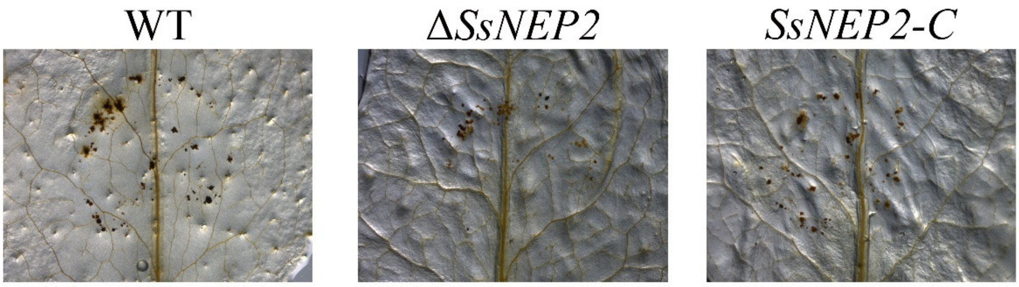

Supplementary Figure S2. Δ*SsNEP2* reduces H_2O_2 accumulation in *Arabidopsis* leaves. DAB staining of *Arabidopsis* leaves inoculated with WT, Δ*SsNEP2*, and *SsNEP2-C* strain after 8 h.

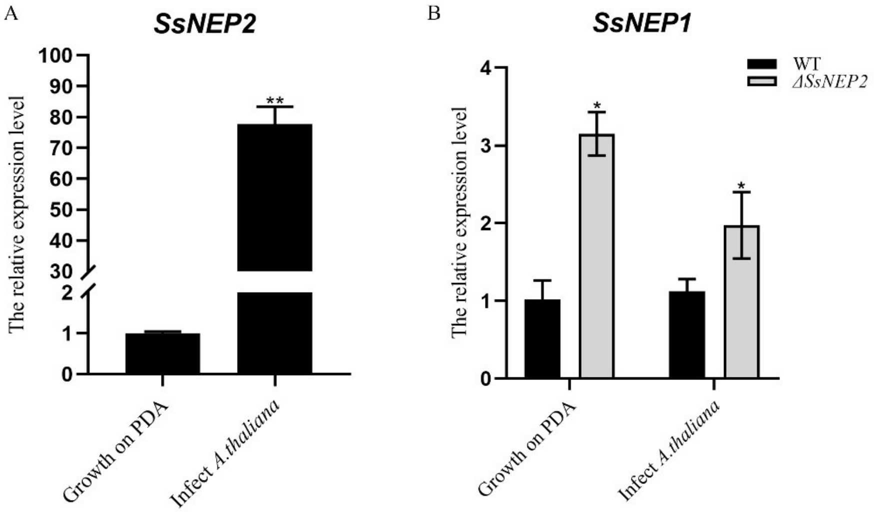

Supplementary Figure S3. *SsNEP2* is involved in the infection process of *S.sclerotiorum.* (A) Relative expression levels of *SsNEP2* in wild type strain grown on PDA or inoculated on *Arabidopsis* leaves after 24 h. Error bars represent SD. Statistical significance was analyzed using Student's *t*-test (* $p < 0.05$; ** $p < 0.01$). (B) Relative expression levels of *SsNEP1* in wild type strain and Δ*SsNEP2* mutant grown on PDA or inoculated on *Arabidopsis* leaves after 24 h. Error bars represent SD. Statistical significance was analyzed using Student's *t*-test, the relative abundance of *SsNEP1* in wild-type grown on PDA was set as one (* $p < 0.05$; ** $p < 0.01$).
